# The Influence of Tropical Climates on European Constitutions

**Published:** 1857-07

**Authors:** 


### NORTH AMERICAN

### MEDICO-CHIRURGICAL REVIEW.

JULY, 1857.

### Analytical and Critical Reviews.

ART. I.—*The Influence of Tropical Climates on European Constitutions, including Practical Observations on the Nature and Treatment of the Diseases of Europeans on their return from Tropical Climates.* By JAMES RANALD MARTIN, F.R.S., Surgeon to the Bengal Army, retired; late Surgeon to the Native Hospital, Calcutta, etc. etc., London, 1856, pp. 599.

THE work of Dr. James Johnson on the subjects treated of by the volume before us is everywhere well known, and acknowledged to be one of surpassing excellence. It was originally published in 1813, from materials collected and arranged by the author during three years service in the Indian seas, he being at that time scarcely twenty-four years of age. Yet the work of the youth showed the hand of the master; its merits were immediately acknowledged by the public as well as the profession, and it is not improbable that the satisfaction so natural to an author to feel, and the gratification of a worthy ambition consequent on this favorable reception, contributed not a little to support him during a severe illness, while abscess after abscess discharged from the liver, and through a long and tedious convalescence from disease contracted in the fulfilment of arduous duties in unhealthy climes. This work passed through edition after edition, until in 1841 the sixth appeared, in the composition of which last, he was assisted by the author who now appears in the field to continue alone the work so ably commenced by his lamented predecessor and companion.

In the preface to the present work, Mr. Martin modestly informs us of his connection with Dr. Johnson, and of the experience which he thinks has fitted him for the task of rewriting the work. Being called upon to prepare a seventh edition of the original work for the press, he felt that it could not be presented as a complete treatise on the subject; additions having been made to every successive edition, consisting mostly of articles from the pen of Dr. Johnson upon different and unconnected branches of the subject, until it had become "in great part a collection of reviews, which, excellent as they were at the time, could not now be made to represent the advancing knowledge of the day." He therefore determined to rewrite and recast the whole work, adding to the contributions of Dr. Johnson, the result of opportunities, both varied and peculiar, extending over a period of twenty-two years' service in India, during peace and war, among Europeans and natives, in hospital and private practice.

If called upon to give briefly at the outset the characteristics of Mr. Martin's work, we should say they are, Firstly, an attempt to base his medical practice upon the sound basis of physiology, and, Secondly, to impress the importance, and point out the best means of preventing disease. Although in the former he is but following a path already travelled by his predecessor, and in the latter is only continuing the work commenced by Robert Jackson, Hennen, M'Gregor and others, yet he deserves no less praise for having ably seconded the efforts made in these observers. Progress in practical medicine will be made in proportion to the more perfect and definite knowledge we obtain of physiology; and the ardent pursuit of the connection between physiology and therapeutics must animate every lover of medical science with the hope of the speedy dawning of better days. On the other hand, we are proud to point to the labors of medical men in the noble work of preventing disease—to which they are animated alone by the spirit of philanthropy,—the progress of which they are forcing in despite of the direct opposition of those in high places, and by means of which they have already conferred immense benefits on the human race. Success in this direction constitutes the best progress of our art, while at the same time the labor attending it is the least rewarded. "In medicine alone," says Marshall Hall, "is improvement without recompense."

In no other circumstances are sound physiological doctrines more necessary to the medical man than when he leaves the temperate zone for the tropics, having under his care large bodies of men, whose health he is to conserve, and whose diseases he is to cure. Without clear ideas of the influence, both primary and remote, which the new external circumstances will have upon the various functions of the body, he will be unable to distinguish those habits of life which will coincide with the climate in producing disease, to institute proper regimen to obviate its effects, or to adapt his treatment to those diseases which are the result of it. The first one hundred and forty pages of the present work are occupied with these subjects; in the consideration of which the author is full and complete. For his definition of climate he adopts that of Cabanis: "L'ensemble de toutes les circonstances naturelles et physiques au milieu desquelles nous vivons dans chaque lieu," and enters into a full consideration of every particular included in so general a definition. Dress, food, drink, exercise, clothing, sleep, and the regulation of the passions, are each treated of; and there are also copious statistics of the mortality of Europeans in India, drawn from the returns of the army and navy, the results of which are compared with those obtained in other portions of the world. Of course an analysis of the climate of different portions of the British Empire in India, is of no interest to us here; nor, indeed, is it worth while to say much in regard to the effect upon the system of tropical climates in general, the work of Dr. Johnson being familiar to most of our readers. We shall allude, therefore, to only a few interesting particulars.

The exceedingly fatal influence of the Indian climate upon European constitutions becomes more evident upon every page of the book, and far surpasses our anticipations, for we had supposed the mortality of those regions due more to sweeping epidemics, and their unhealthiness to causes against which some years' residence gave comparative security, rather than to the slow unceasing influence of a climate which no acclimation overcomes, and which seems in a league of unending hostility with the native against the foreigner who has invaded his shores. On this point our author quotes Twining, who says that, after careful inquiry, *he was not anywhere able to find a sample of the third generation from unmixed European stock*, and adds:

"I believe that such is scarcely to be found in any part of India, and least of all in Bengal Proper:—so much for the question of European colonization, respecting which much has been said and written—regardless of the fact that Nature has set her ban—a blighting interdict upon it."

Less surprise will perhaps be felt at such a statement after reading of the influence of the climate upon the inorganic world.

"A familiar, but emphatic illustration of the effects of our climate may be seen in its influence on the habitations of Calcutta. Constructed of the finest known materials, whether of wood or mortar, and of such solidity that in England they would endure for centuries, and in Upper Egypt for a thousand years; they are here, through the destructive influence, and severe alternations of climate alone, rendered in a score of years, or less, fit habitations only for crows; in much less time indeed, they may be seen reduced to a heap of rubbish covered with vegetation. 'A deserted village is overflowed by the forest like the waves of the sea, in the course of two wet seasons, and the traces of man are buried by the exuberant productions of nature'"

The only class of persons to whom the climate seems beneficial are those in the early stages of consumption:

"There are two classes of persons to whom our climate seems genial—the weak-chested, as they are called in England, who are of a scrofulous habit, but in whom pulmonary disease has not actually declared itself. These are saved by going to India; and I have known many persons in the curable stage of consumption—that is, laboring under the preceding stage, or that of 'tuberculous cachexy'—enjoy good health in Bengal, and survive their brothers and sisters at home. The fate of those, on the other hand, who go to the East with suppurating tubercles, or even in the stage approaching it, is only precipitated."

Returns of the French army in Algeria are then quoted, showing the rarity of phthisis in that climate, where only thirteen cases were reported out of a total of 8485 patients, and but ten deaths from pulmonary disease out of a total of 871 deaths; and the following conclusions are arrived at:

"It may, then, be asserted, on the ground of statistical evidence of the most extended nature, that climates and employments that induce sweat, or that induce a gentle perspiration not subject to check, are unfavorable to the existence of scrofula or consumption. It may be inferred, also, that the curative virtue of marshy countries, as respecting phthisis, resides less in the miasmatæ themselves than in the uniformity of temperature, the heat of the atmosphere, a moderate degree of moisture, and the absence of dry, sharp winds."

The chapter on The Prevention of Disease is full and comprehensive, containing remarks upon every particular relating to hygiene. We have already alluded to the prominent position which this subject holds in the work—a position in accordance with the motto which is placed on the title-page: "Rerum cognoscere causas, medicis imprimis necessarium, sine quo, nec morbum curare, nec præcavere potest;" and in the course of the article upon this subject, we find the following paragraph:

"It is justly observed by a popular writer, that there never were any specifics discovered against the plague, the sweating sickness, or the leprosy; and yet the leprosy, the sweating sickness, and the plague, are now among the things unknown to us. They disappeared, not before any marvels of medicine, or any perfection of chemical science, but before the gradual amelioration of our condition through sanitary improvements."

There is great care taken to point out the influence of length of residence in the climate in modifying regimen as well as the treatment of disease. Thus the author warns the new-comer against those condiments and stimulants which the old resident takes, not only with impunity, but with benefit: in the one they produce highly deleterious effects upon the digestive organs; in the other, they serve to rouse the stomach from the torpor which is one of the consequences of long-continued heat. Believing, too, that the use of stimulating drinks, as a means of "keeping cool" in hot climates, is as prejudicial to health as it is opposed to sound physiological teachings, he agrees with Dr. Johnson in inculcating total abstinence doctrines, to the new-comer at least, and is sarcastic upon those medical writers who have recommended stimulants as a means of "supporting perspiration." His testimony upon this subject is worthy of reception, even if it were not supported by our knowledge of the functions of the body and the effect of alcohol upon them. There are not wanting physicians in other climes than India who recommend stimulants as prophylactics against extreme heat:

"We hear much amongst vulgar habitual topers of the supposed prophylactic influence of spirits and cigars against night exposure, malaria, and contagion; but no medical observer, in any of our numerous colonies, has ever seen reason to believe in any such delusive doctrine. Nor is there in reality the smallest foundation for it. All excitement is followed by corresponding depression of the vital functions, and it is then that the toper is doubly liable to suffer."

"It may be received as a truth, that during the first two years of residence at least, the nearer we approach to a perfectly *aqueous* regimen in drink, so much the better chance have we of avoiding sickness; and the more slowly and gradually we deviate from this afterwards, so much the more retentive will we be of that invaluable blessing, HEALTH."

The author contrasts the course of the European, resorting to brandy and water to support the extreme heat, with that of the native, who retires to the inmost apartment of his habitation, and drinks cold water or sips sherbet. For himself, he found warm tea the most refreshing beverage after the most severe marches in the sun; and he cites the just physiological course pursued by Napoleon to obviate and remove the effects of extreme fatigue—a warm bath and a cup of strong coffee. In this connection he alludes to "the recent luxury—the American ice—which has now become an article of necessity in the East," and thinks that those who have once experienced its beneficial effects will "need no arguments in favor of promoting so remarkable an instance of American enterprise."

We cannot avoid citing two instances in which the most fatal results have attended military expeditions undertaken and carried on in defiance of medical advice. Although more properly belonging to the military and naval surgeon, they cannot but prove interesting to all branches of the profession, for the disaster was so plainly foreseen in each case by medical men, their advice so contemptuously rejected, and the penalty which followed so speedy and so severe, that it seems as if they might stand as warnings for all future time.

The first was the expedition to the Island of Walcheren, in 1809–10. It was one of the largest expeditions which up to that time had ever left the shores of England; consisting of 70,000 men, in good health and well equipped. The landing took place on the 31st of July and 1st of August, and by the 10th of October, 587 per thousand of the strength had fallen sick, while there died 142 per thousand! Yet the malarious nature of the climate had been described years before; it was well known to Napoleon, whose tactics were to keep the enemy in check, trusting to the fever to destroy them; and destroy them it did. The expedition miserably failed, and entailed upon the British nation an annual tax of one million pounds sterling *forever!*

The expedition to Rangoon was equally disastrous, and the mortality equally unnecessary. During the first year of the war there perished of the British force 48½ per cent., of whom only 3½ per cent. were killed in action, or died of their wounds; and the total European loss during two years of this Burmese war was 720 per thousand of strength, "being the greatest mortality of which there is any record." Here, besides the evil influences of one of the most deadly climates, the diet of the men consisted alone of biscuit and beef, which latter had been slaughtered and packed at Calcutta during the hottest season, when putrefaction commenced before the salt could penetrate it! Seeing the inevitable consequences of such combined deleterious influences,

> "The superintending surgeon of the division warned the officer commanding that, without fresh animal food and vegetables the European soldiers must perish from scurvy. The answer was characteristic; 'Medical opinions are very good, sir, when they are called for.'"

But there has been a more recent instance than either of these. The scorbutic dysentery of the Crimea destroyed more than the bullets of the Russians, and was brought on in spite of warnings, such as these we have quoted; in spite of medical opinions given on the spot. We confess that this compels us to doubt whether the time has yet arrived when the "how-not-to-do-it" system will be abolished, although our author is pretty sanguine on the subject. He thinks that,

> "Humanity is now at length pretty sure to have a powerful press on its side; and no governor or commander, by sea or by land, can any longer venture to do, or to leave undone the things which I have seen done and left undone."

The most frequent causes of death in hot climates are fevers, diseases of the liver, and dysentery. It is to these subjects we turn with anxious interest to the writings of one who has spent the greater part of his life in treating them, where they are most prevalent and most severe. Those who see in the prairies and in the valleys of our Western States the chief habitation of malaria, have but a narrow view of one of the most wide-spread and destructive causes of disease. There are persons who, having spent a few years in practice in some locality a little more than ordinarily malarious, imagine that they understand the whole matter, and fill pages of our journals with effusions upon a subject of which they have obtained but a limited view. If we would see malaria at *home* we must go, as we would to see the king of beasts, to the tropics, and there we shall find it with unshorn strength, virulent and powerful beyond anything we had anticipated. Under the long-continued heat of the tropics we find, too, inflammation and abscess of the liver occurring in frightful frequency; and dysentery, whether cause, consequence, or companion of these, fatal beyond anything seen in more temperate regions. All these being forms of disease not uncommon among us, and some of them not infrequently prevailing as epidemics, it is but judicious to examine with care the opinions of those who have had much experience with them, in regard both to the causes which give rise to them, and the principles which should guide us in their treatment. This we propose briefly to do in reference to a few points of such an extensive range of subjects, that those who wish may compare the doctrines and treatment of our author with those of Copland, Johnson, Twining, and Budd; or gain additions in practical knowledge upon a class of diseases which differ more in their grade than in their nature, from those of our summer and autumn seasons.

One of the most interesting subjects is brought before us soon after opening the work; that yet unsettled one, the *origin of malaria.* What is it? Whence does it come? These are questions which yet remain unanswered, although so frequently attempted. Our author adds another to the list of those who have made the effort; not, however, we are happy to say, with that tone of confidence which betrays self-esteem, and proclaims a mind bowing in idolatrous worship before its own theories, but in the suggestions which become the man of science, throwing out his own observations as nuclei, around which those of others are to cluster. He became satisfied very early in life that the geological nature of the soil is a most important element in physical climate, and he thinks he has found in a ferruginous soil and a high temperature the most important elements for the production of malaria. He states his experience in India; and diagrams are given showing that wherever malarious fevers are most deadly, there ferruginous soil is invariably found. He quotes from other army surgeons there in favor of the same view, and who believe with Watson, that the theory of the vegetable or marshy origin of malaria cannot be maintained. Not content with India, he sent for and obtained specimens of the soil from all the unhealthy stations on the coast of Africa, and from various parts of the world; all these, upon analysis, yielded a large amount of ferruginous matters. Upon this subject he makes several allusions to this country concerning the doctrine he has broached. He quotes the "Excursions" of a writer who constantly mentions "malaria" in connection with ferruginous soils, with red sandstone rock, and with "the red mud of the Arkansas;" and states, on the authority of "Dr. Forey," that the difference in the composition of soil on the sea-coast and in the interior of the United States, causes the vast difference observable in the prevalence of intermittent fever. From M. Baudens, surgeon-general to the Military Hospital at Versailles, he quotes at some length on the influence of soils on health. This writer says that from the similarity of geological composition of the soil, we can predict the character of the predominant diseases in two regions, however remote from each other, and continues:

"Finally, as, under a dissimilar geological constitution, we see that the forms of disease differ entirely at the mouth of the Po from those of the Arno, so we can predict a similar difference between the eastern and western coasts of America, and affirm safely the rarity of marsh fevers at the gigantic embouchures of the Simpson, the Columbia, the Oregon, and the St. Francis.'"

We introduce this for the benefit of our professional brethren on the Pacific coast, and look to them to decide whether the statement of M. Baudens can be maintained. The question is an interesting one, and should be borne in mind by all in making observations on the subject, since the present theories do not cover all the facts in the case, and it becomes us to investigate every new proposition, until, finally, we attain the truth.

But our author does not see in malaria the sole cause of disease; there is another agent, which, although its effects are better measured by physical instruments, is as subtle in its essence as the supposed emanations of marshes:

"It has long been known, that animals waste and perish when they have been deprived of their positive electricity, by being attached to the opposite pole of a galvanic battery. When the human body, on the other hand, has been for some time exposed to an atmosphere of a negative electricity, it is believed by many to become thus incapable of resisting the various causes of disease, as the exhalations from the earth, the force of epidemics, &c. The varying state of the earth's magnetism cannot fail of exercising powerful influences on human health, however little may be the present amount of our knowledge respecting the nature and operation of these influences."

The first disease treated of in the work is the Remittent Fever of Bengal. The author commences with a history of the disease from the first intercourse of Europeans with India, and shows a striking decrease of mortality from what formerly prevailed. After careful examination, he attributes the devastating fatality of former years to the complication of a scorbutic element, and concludes that the decreased proportion of deaths has arisen more from improved hygienic regulations than from improved treatment. He thinks that but little success would now attend efforts to cure Europeans laboring under the combined influence of concentrated malaria and scorbutus. Without being prepared to assent fully to the statement of the author, that remittent fever is "the most prevalent form of disease known to the world, and that by which it is supposed the greater portion of the human race is prematurely destroyed," we know it is a form of disease which comes under the care of the practitioner in many parts of this country, more frequently than any other during some months of the year; and as we cannot find any difference between it, as described by Mr. Martin and as seen in our own country, except in the far greater severity of the former and its more frequent complication with abdominal and cerebral lesions, we shall try to glean what we can from his chapter on this subject for the benefit of our readers.

The milder forms of the disease need not detain us; they are exactly similar to those in our own country; but they are rare there, and what is here the exception, is there the rule. Those forms of the disease accompanied with complications of the abdominal or cerebral organs and with a tendency to collapse, are there most frequently seen, and appalling examples are given of the suddenness of their attack, and the rapidity with which a fatal termination has followed. In the great majority of cases, the seat of the "congestion" is the abdomen—the stomach, duodenum, and liver being found, after death, "turgid, of a dirty red color, and softened," or even the mucous coats ulcerated; in more rare but more frequently fatal cases, the brunt of the disease falls on the brain, and serous effusion is found in the ventricles; the result of vascular determination, "which may pass on to congestion, or inflammation and its consequences." These complications may attend the case from the beginning, or they may arise at any period of its course, so that the physician is warned to be "ever expecting the unexpected!" The tendency to collapse is extreme, and the most alarming prostration of the vital functions may take place even within a few hours.

"The depression caused by a violent blow on the abdomen, more nearly resembles the febrile collapse than any other morbid condition with which I am acquainted; and both, probably, depend on a disturbance in the functions of the organic nervous system—the powerful though silent source of many symptoms known to us only by their effects."

There is also a great tendency in this fever to establish organic disease of the viscera of the abdomen, and what is so rarely a sequence of the disease here, is scarcely guarded against there; and this peculiar liability must be kept prominently in mind, in order to do full justice to the patient.

"It must be held in recollection, too, that what are termed congestions, subacute and acute inflammations, tend firstly, to destroy life speedily; and secondly, that, in their remoter results, they found organic diseases which have the same eventually fatal effects. In either case they form conditions of the utmost importance; for, in the first instance, the immediate cure is involved, so as to save life; in the second, the prevention of disease, more or less deadly."

In the selection and application of the means of cure the greatest stress is laid on the different modifications of the disease, the effect of different localities, seasons, and epidemics, and on the proper adaptation of these means to the constitution of the patient, and stage of the complaint. This is the only course pursued by the scientific physician, and we mention it to show that our author belongs to that class, and not to those who have found some new and wonderful mode of cure, equally efficacious in every form and stage of a complaint. He shows by an interesting history of the treatment of those fevers what a baneful influence such writers as these have exerted; that "at different times bark, calomel, and bloodletting, have each in its turn been in almost exclusive favor," but he concludes with the very evident proposition that,

"It is only by a rational and well-adjusted plan, which shall call into operation all the aids suggested by science and by experience, that such a disease as fever can anywhere be justly treated."

He considers the first indication to be the reduction of the force and frequency of the arterial action; "which if allowed to go on unrestrained, would injure or destroy some organ essential to life." To fulfil this indication, there is but one remedy prompt enough to be of value—bloodletting, —and we find our author a warm supporter of depletion, in what we call "congestive fever." He says it "simplifies all the subsequent means of cure;" and we might quote largely from him in favor of this practice, as showing that the mortality has always been greater; and especially that serious abdominal diseases have been more frequent when it has been omitted; and that even the treatment by bark has been of no avail when not preceded by depletion.

"I am aware that a kind of fashion is at present moving against the practice, almost to the desire for its total exclusion; but those who are acquainted with the history of medicine, and who have seen and understood the acute nature of tropical diseases, will take fashion here at its proper value and no more."

But to repeat, depletion is not to be blindly pursued, but the greatest care is taken to point out that it should be "*always practised at the very onset of the stage of reaction*," and that "*what was a saving means at the commencement of the paroxysm, is as surely destructive at the end of it.*"

It is evident that the practice of bloodletting in fevers must rest upon very different grounds from what it does in inflammation, and we regret that we cannot here examine the subject at length. The first question to be decided is the extent to which *congestion* influences the fatal result. If it is not the principal evil, as held by Wood, in his "Practice of Medicine," but that we must look upon faulty innervation as the source of danger, then it is clearly contra-indicated and must necessarily be injurious. But that congestion is the principal danger in many cases, is the conclusion we draw from the author before us, and it is supported by what we have seen of such cases; but he clearly points out those cases attended with great depression of the vital functions, as unadapted for the use of the remedy. It is not alone to severe cases, however, that bloodletting is restricted; he is a warm advocate for its use in almost all cases of remittents and intermittents; believing that it prepares the system for the more speedy influence of all other remedies, shortens the course of the disease, and is highly efficacious in preventing those visceral engorgements so frequently following malarious fevers. In reviewing our treatment of some severe cases of remittent fever, and some obstinate intermittents, we have regretted the omission of this remedy, and more readily resort to it now in the early stages of the disease.

The most reliable remedies after depletion are mercurial purgatives and the sulphate of quinine. The former "rouse the dormant sensibilities of the bowels, and aid in restoring the balance of the general circulation, especially when bloodletting has preceded their administration so as to remove congestions, and thus afford a renewed activity to the functions of secretion;" they "relieve the portal circulation from stagnation, and the liver from consequent disease." The latter, he says, should not be used, unless in the severest cases, before depletion and cathartics have overcome the state of congestion and of disturbed and impeded secretion. In this he is supported by Annesley, Robert Jackson, and Copland. The neglect of these preparatory measures entails visceral disease upon the patient if he escape the attack of fever.

"The nearer the patient can be brought to the state of remission, the surer will be the operation of the antiperiodic."

"The random exhibition of quinine by timid, careless, or inexperienced practitioners, does infinite harm; and in former times, when bark alone was relied upon for the cure of all tropical fevers, the results were horrible."

"Where the remissions are becoming sufficiently well-marked, quinine should be given in full doses, without waiting for a perfectly favorable condition of the system; in bad fevers, we should seize the very first dawn of remission to exhibit quinine. Some practitioners recommend that before this drug is used, we obtain a clean tongue, natural secretions, and the absence of all heat of skin, and of local complication. I believe this to be a very dangerous practice: if we are to wait for everything favorable, we shall often have to wait too long, and till it has become too late."

* * * * "I have always given quinine, in more favorable circumstances, in disregard of certain abdominal complications (those of the cerebral cavity should, in general, exclude its use), believing that if I arrested the paroxysms, the progress towards curing the disease as a whole, greatly outweighed any harm which the quinine could possibly do to the local affection, the treatment of which by local depletion, by mercurials, or by counter-irritants, is not interfered with by the means in question. Again, all tenderness on pressure, or local pain, does not necessarily constitute inflammation; and even where such is actually present, its character is often so modified by malarious influences and anæmic states of the general habit, as to constitute a state very different from idiopathic and uncomplicated inflammations."

Doses of five grains are preferred by the author, and a favorite mode of exhibition with him is to give it in combination with the cathartics belonging to the early treatment:

"Given in this manner, the quinine applies itself to the whole extent of the mucous digestive surface, so as to give full effect to its tonic and antiperiodic influences; and a larger dose of it can thus be borne in the early stage of fever than if given without the purgative."

These are the principal remedies in the treatment of remittent fevers everywhere, and those upon which the only reliance is to be placed in severe cases; other remedies are but adjuvants, and although he considers all of them, he places no reliance on them. Emetics he is not as partial to as are European practitioners in general, judging from the compendium he gives of treatment; he thinks we cannot yet distinguish the cases in which they will prove beneficial from those in which they will be injurious. No mention is made of washing out the stomach with copious draughts of warm water or herb tea, which is of essential service in allaying irritability of the stomach in our remittent fevers, although he states vomiting to be a frequent and distressing symptom.

Mr. Martin awards priority to Robert Jackson, instead of Dr. Currie, of Liverpool, for the introduction of warm and cold effusions in the treatment of fevers. He relates cases where the latter were of essential service, yet thinks they are too dangerous to be generally resorted to in severe forms of disease. "Measures difficult of application, and sometimes doubtful of result—excepting when used with extraordinary tact—will not be generally adopted or employed by ordinary medical practitioners."

Diaphoretics he looks upon as valuable in this fever, but in the severe cases there is not time for their action.

Wine and spirits are only of value in cases of collapse; then they are "invaluable, and should be always resorted to."

We may add, that mercury should never be carried to the extent of affecting the system, unless some important organ is congested or inflamed; then it is to be given in small and repeated doses, combined, by preference, with camphor in most cases; "it repairs mischiefs which no other means with which we are acquainted are capable of touching."

Dysentery is so peculiarly a disease of hot climates, and so much more severe and frequent in them, that we derive our most valuable information concerning it from those writers who have seen it within the tropics; we may instance Annesley, Copland, and Johnson. As occurring in India, it does not present any great peculiarities distinguishing it from the disease elsewhere; it is more severe and fatal, and the mortality occasioned by it is appalling; rendering it well deserving of the name of "scourge of armies." Complications, especially hepatic, are more frequent than in temperate regions; but its cause and treatment, when occurring in scorbutic diatheses, are not of sufficient interest to detain us here. The symptoms, course, and nature of the disease are well known. We pass to consider the treatment, in which, if we find nothing new, we may find confirmation of the usual course prescribed, by one who has had most abundant facilities for observation. Early and prompt attention to the patient is shown to be of primary importance: "Time is either our most important ally, or else our most powerful enemy;" and it is urged that "in every case dysentery must be speedily overcome, if we would save our patient."

"It will be found with dysentery, as with fever, that a sufficient abstraction of blood by venesection, practised at the very onset of the disease, will simplify and render easy all the subsequent stages of the cure; so much so, that in mild and uncomplicated dysentery, as in the violently acute and inflammatory type of the disease, bloodletting is the primary as well as the cardinal measure. In acute uncomplicated dysentery, a moderate bleeding from the arm, followed by a full dose of calomel and James's powder, or that of ipecacuanha, say twenty grains of the latter to ten of calomel, given at bedtime, a warm bath or hot fomentations to the abdomen—a morning aperient, followed during the day by sudorifics conjoined with diuretics—the moderate use of demulcent drinks, allowing no other sustenance—will, in a very few days, bring about convalescence."

This is a compendium of Mr. Martin's treatment, varying with the varying aspect of the disease. He warns the young practitioner against model plans of treatment, and acknowledges his inability, in an ever-varying disease, to lay down "any catholic rules of treatment which shall be suitable to every case." Local bleeding should follow general, if there be pain or tenderness in any part of the abdomen in the early part of the disease; and blisters later. The continuance of mercurials are to be judged of by the tongue and discharges; but they are by no means considered indispensable, except in hepatic dysentery. Opium, combined with ipecac, is the great remedy after the inflammation has been subdued by depletion. In the latter stages of the disease—

"When soreness along any portion of the large intestine exists, with a suspicion of ulceration of the mucous membrane, the use of washed sulphur alone, or in combination with the bi-tartrate of potash, will prove the best aperient given every other morning."

In this stage of the disease, enemata are recommended—simply anodyne, or anodyne and astringent, containing eight to ten grains of acetate of lead, and two of opium, in cold water, if the patient will bear it. The latter remedy is also recommended for tenesmus when distressing. The natives of India, we learn, are singularly averse to injections; nothing will induce them to consent to their use—"the very impression of death making no alteration in their fixed aversion to every such remedy."

In dysentery complicated with hepatic disease, the treatment is essentially the same; depletion being persevered in until "we remove congestion of the portal system, while by the nightly use of calomel and ipecac, followed by compound powder of jalap in the morning, with diaphoretics and diuretics through the day, we relax the skin, emulge the liver, and restore the lost balance of circulation.

In hemorrhagic dysentery, the most powerful astringents in the largest doses are recommended in union with opiates. The author is very partial, in this form of the disease, to acetate of lead, which, he says, "may be given more liberally and more safely than is generally supposed." Among English practitioners, it may be necessary to urge an increase of dose of this medicine; but we do not think such advice needed here!

"In hemorrhagic dysentery, the lead should be given every two hours, so as speedily to establish its influence, while, to each dose, from half to a grain of opium is added, using, at the same time, strong enemata of acetate of lead with opium, thrown with sufficient force to enter the curve of the colon; and this course of treatment must be persisted in, till the discharge of blood shall have totally ceased."

The complication of the disease with malarious fevers, is that most common in this country, and we copy his remarks on the subject:

"In treating the complications of dysentery with fevers, remittent or intermittent, we must have regard to the type and prevailing character of the febrile movement, as well as to the paroxysms, in addition to the attention that may be demanded by the condition of the bowels. When the fever is of an ardent, inflammatory character, our antiphlogistic means must be applied with an energy proportioned to the violence of the disease; while in the low adynamic type we must have recourse to bark, quinine, opiate, chalybeates, the vegetable and mineral acids, improved diet, and change of air."

In regard to the treatment of convalescence, he thinks he cannot impress sufficiently upon the inexperienced, the necessity of caution, and that too much attention cannot be paid to prevent a relapse. He adds the following important remarks:

"In convalescence from dysenteries, acute and chronic, much active benfit will be promoted by the external and internal use of the nitro-muriatic acid. When the spleen is found to be enlarged, whatever the stage of dysentery and whatever the type of the fever, mercury must be carefully avoided, trusting to the means just named (in the preceding quotation), and to all such as vivify and improve the blood. It is almost unnecessary to add, likewise, that the abstraction of blood, in the complication of these fevers with dysentery, should be practised only when the fever is of the acute or ardent type, the adynamic forms, as well as the splenic cachexia, not admitting of the use either of mercury or of bloodletting."

Finally, as in fever, he gives a compendium of the treatment of the disease, from Bontius, in 1622, down to Copland, in 1835; and he reviews sharply those pet systems of practice, which are confined to the administration of a single remedy, or class of remedies; and which have been lauded by their originators as unfailing, but have been rejected by the profession as unscientific, or proved by experience to be inefficacious. Such is the treatment by bark or quinine alone; that by ipecacuanha; the repeated strong purging of Twining, and the ipecacuanha, and extract of gentian; and that by solutions of the purgative salts, and tartar emetic. We need not quote his remarks on each of these modes of treatment; the following quotation will serve at once to show his appreciation of them, and the scientific view he takes of the adaptation of treatment to the case:

"He who would treat dysentery with success, while he shuns exclusive means, must assign to each remedy its proper value, and neither more nor less. Bloodletting, sudorifics, and purgatives constitute the most universal remedies, and in simple uncomplicated dysenteries they will prove all-sufficient. But when the abdomen is tumid, and there is pain in the liver, or in any other region, while the nature of the discharges indicates advancing inflammation, calomel conjoined with sudorifics, and repeated to meet the occasion, will powerfully aid the curative effect, through its influence on the depurative functions; on the circulation, in unloading, jointly with purgatives, the gorged vessels of the abdominal organs; on the blood and on secretions generally—and on the very sudorific function which we so much desire to excite. This, in so many words, I believe to be the real value of calomel in the treatment of dysentery; and the inexperienced have been led into much unnecessary doubt, and into no small amount of error, by some writers who regard calomel as a remedy of necessity, and by those who, on the other hand, would exclude its use altogether.

"It is hardly necessary to repeat that while calomel is a most powerful agent in its place, as an aid to bloodletting, the pushing it to the extent of ptyalism is here by no means intended; nor should mercury in any shape be used in adynamic forms of the disease; in the splenic cachexia, nor in states of anemia; for in all these conditions of the system its actions are most injurious."

Hepatitis is considered under two heads, as the inflammation is adhesive and superficial, or parenchymatous and suppurative. The latter is not only more important from its termination in abscess, but from the frequency with which it pursues its course without any symptoms to give warning of its presence. "It is one of the most dangerous of diseases, because of the total absence of urgent symptoms, and its consequent insidiousness; the process which leads to destruction is here silent and rapid."

The question of the connection existing between hepatic abscess and dysentery, the author leaves as he found it, an open one. His testimony agrees with that of many other writers upon the subject, without any new facts being added to substantiate it.

"We know not, in too many instances, which of the diseases is the primary,—that in the bowel or the liver. But there are cases, and they are numerous, in which we can plainly discern hepatic disease and hepatic abscess as primary, concomitant, and secondary affections in the time of their occurrence; but whether they are to be regarded as a cause, a concurrence, or a consequence of dysentery, is not so easy to determine."

Nor does the author determine it; he quotes from a writer in the Lancet, and from Dr. Henoch, in the Medico-Chirurgical Review for July, 1854, in favor of the connection of the two diseases from their origin in the same causes, and not from any mutual relation existing between them. This is the view held by Annesley,* whose work on Diseases of India stands in very high repute; and we find it supported by Waring,† in his work entitled "An Inquiry into the Statistics and Pathology of some points connected with Abscess of the Liver, as met with in the East Indies." He bases his arguments upon the statistics of the two diseases.

It is seen then that the majority of those who have seen diseases of the liver and dysentery in tropical climates dissent from the opinion of Dr. Budd, which is, that the hepatic abscess arises from phlebitis, or from contamination of the portal blood from absorption of septic matters from the intestine. But that author deserves no less credit because he did not see the whole truth; in the amount of information collected in proportion to the field of observation, no one has surpassed him, and in his admirable treatise he has done infinitely more to advance our knowledge of diseases of the liver, than many who have written from the full wards of tropical

* See Budd on the Liver, 2d edition, London, 1852, p. 78.

† Medico-Chirurg. Review, July, 1855.

hospitals; but only those who have heard him lecture, and have seen him at the bedside, can fully appreciate the acuteness of his powers of observation, the clearness of his descriptions of disease, and the skill with which he adapts remedies to the case in hand.

It cannot but be a matter of deep regret that a work, so full and complete in every other respect, should fail in one point, where we look with the greatest interest for information; the diagnosis of hepatic abscess is a question that we are obliged to refer to such men as the author. Yet we have here but a few lines more than a page upon this point; hectic, rigors, profuse perspirations, and, "when the abscess is large," enlargement of the side, are mentioned; and we are informed of the great obscurity, both of degree and manner, in which the symptoms are displayed; but no pains are taken to point out the relative frequency and value of these signs, to separate the local and the peculiar from those more general ones, which may belong to suppurative disease of internal organs in general; statistics are lacking just where they are most desirable. In this respect there is a great contrast between Mr. Martin and Mr. Waring, whose work, already alluded to, notes the relative frequency of every symptom. We extract a few of the more important results.

| | | | | | |
|---|---|---|---|---|---|
| Pain in hepatic or epigastric region | occurred in | 153 | cases | out of | 173 |
| " right shoulder | " | 48 | " | " | 76 |
| Enlargement or fulness of side, | " | 90 | " | " | 101 |
| Vomiting and nausea, | " | 74 | " | " | 84 |
| Jaundice, noted in only | " | 9 | " | " | 360 |

A reviewer, Dr. Parker, thinks it probable that this latter symptom was undoubtedly absent in the cases where it is not mentioned, and states, that in twenty cases observed by him, jaundice was present in but one.

In regard to opening abscess of the liver, he agrees entirely with Budd, and states that he has often seen it done, and performed it himself, but "never saw it result in any eventual good." He appends a table of fifty-seven cases in which the abscess was punctured; death took place in forty cases, only seventeen recovered. Mr. Waring mentions eighty-one cases punctured, in India, during the present century, but whether these include Mr. Martin's, or not, we cannot say; of these, sixty-six died, or nearly 81½ per cent. Both these authors give the following table of the proportion of cases in which a plurality of abscesses, or concomitant ulceration of the intestine was present, thus precluding the probability of recovery.

| | |
|---|---|
| Total number of cases, . . . . . . | 300 |
| There were plurality of abscesses in . . . | 108 |
| | 192 |

| | |
|---|---|
| Of 177 cases, in which abscess was solitary, there was more or less extensive ulceration of the large intestine in . | 76 |
| | 116 |

"Thus, out of the whole number, only in 116, or little more than 1 in 3, could the operation have been undertaken with any reasonable probability of success."*

We find no mention of Graves's method of opening the abscess, after a preparatory incision has been made.

But little general treatment is advised for those suffering from hepatic abscess. The nitro-muriatic acid and bitter tonics, are the chief remedies; and favoring the efforts of nature by gestation in the open air, change of climate, &c., are strongly recommended. The use of mercury in this condition is most strongly deprecated. As it aggravates the irritative fever, and its specific influence favors suppuration and increases debility, it "is always highly injurious."

The table already given is sufficient for prognosis, but surprising and unexpected cures sometimes take place by the efforts of nature alone, and the following remarks upon the mode of termination, are interesting:

"Of the instances of recovery within my personal knowledge, by far the greater number were amongst such as had discharged the pus by the bowels; and one married lady, I remember to have recovered health rapidly, after vomiting the contents of an enormous abscess. I also saw four instances of recovery where the abscess had opened into the lung. Of those in whom the pus was discharged through the external integument, the majority have, within my knowledge, died."

Mr. Waring's statistics upon this point show the mode of recovery to have been as follows:

| | |
|---|---|
| By the abscess opening into the lung, or the pus being discharged by expectoration through the bronchi, . . . | 10 cases. |
| By abscess opening into colon or some part of intestine, . | 7 " |
| " one " " " " and one into the lungs, . . | 1 " |
| " abscess discharging its contents into abdominal cavity, . | 3 " |
| " elimination of pus by kidney, . . . . | 2 " |
| " spontaneous absorption, . . . . . | 2 " |
| | 25 |

Dr. Parker thinks the diagnosis of the three latter heads very doubtful. He is also of opinion that the great relief experienced from opening an hepatic abscess should have some weight with us in considering the propriety of an operation.

We have said nothing of the treatment of acute hepatitis proposed by our

* Medico-Chirurg. Review, July, 1855, p. 9.

author; bloodletting and mercury are the chief remedies, and the one is useless without the other. Although he considers at length the use of mercury in this disease, and argues its value from clinical experience, there is a question of interest which he has not touched upon. If mercury is a special stimulant to the liver, why diminish it when this organ is in a state of acute inflammation? How do we reconcile the administration of the same remedy to correct a state of torpor of an organ, and to cure it when in a state of active hyperæmia? These questions have been propounded by enemies of the science of medicine, and they claim to see in this similar treatment of two opposite conditions by the profession, a course inconsistent with sound philosophy. The best explanation we find in the work of another writer* upon diseases of India, whose views upon tropical diseases coincide very closely with those of Mr. Martin. Viewing inflammation, according to the most recent doctrines, as an altered state of the nutritive processes of the part, he concludes that the capillaries concerned in inflammation are only such as circulate arterial blood for purposes of nutrition. It is the capillaries of the hepatic artery, therefore, which are concerned in inflammation, and not those of the portal vein. By this view of the case, the writer reconciles the seeming violation of the general therapeutic principle, since the secreting capillaries are not the capillaries concerned in inflammation in the liver, although in inflamed glands generally, the reverse is the case. In this manner, too, he accounts for the fact that the liver is but slightly enlarged when inflamed, compared with what it is when congested (an engorged condition of the portal capillaries), and thinks it explains why it is that in hepatitis the secretory function of the liver is but little deranged. We have but sketched his arguments and conclusions upon a point so interesting as tending to elucidate what might appear contradictory between pathology and therapeutics.

In regard to the sequelæ of fevers, our author has had a wide field for observation, as must have been evident from his remarks upon the frequency with which the fevers of India are followed by visceral engorgements. In chronic enlargement of the spleen, brisk purgatives, quinine, chalybeates, and stimulating liniments, with change of air, are recommended. The following formula of Mr. Shulbred is given, as having been a favorite remedy in Bengal for half a century:

Pulveris Jalapæ;

" Rhei;

" Colombæ;

" Scammoniæ;

Potass. Bi-tartrat. āā ʒj;

Ferri Sulphatis, ʒss.

* Clinical Researches on Disease in India: by Charles Morehead, M.D.; British and Foreign Medico-Chirurg. Review, January, 1857.

Of this, sufficient is to be taken to move the bowels twice or thrice daily. Twining recommends a similar preparation in a liquid form—the scammony being replaced by ginger, and Tr. Sennæ fʒiv and Aq. Menth. f℥x, added. The following formula of the author's, we think as likely to fulfil the indications : "Saturated solution of sulphate of magnesia, f℥viiss; dilute sulph. acid, f℥ss; sulphate of iron, sulphate of quinine, of each ℥j. Dose: a tablespoonful every morning, or sufficient to produce two evacuations daily."

Where severe irritation of the digestive mucous membrane prevents the employment of these remedies, the author has used pure chalybeates, croton-oil liniments, and warm baths, after the irritation has been allayed. There is no mention of the external application of iodine, which in these cases acts both as a promoter of absorption and a counter-irritant. Two cases of enormous splenic enlargement are reported cured by the iodide of lead, administered internally and externally, and in one of them the remedy "was persisted in to the extent of producing lead colic."

"Iodide of potassium, warm baths, exercise in the open air, stimulating frictions to the affected region, are all beneficial; but in the chronic enlargement of the spleen, with cachectic states of the system, blisters are apt to slough; and as to leeches and mercurials in such instances, they are certain death."

In cases where the liver is involved in disease along with the spleen, the persevering use of the nitro-muriatic acid bath is recommended, with the internal use of the same acids, in conjunction with bitter infusions and aperients. "Here, owing to the hepatic complication, quinine and chalybeates prove ineffective to the cure of the splenic, while they injure the hepatic disease." The nitro-muriatic acid bath is looked upon as a powerful remedy, and especially adapted to those anæmic and cachectic states of the system in which mercury would be highly prejudicial. Its application does not interfere with the use of chalybeates or bitter tonics, and the fact that neither when exhibited internally or externally is its influence in promoting the secretions interfered with by opium, renders it a valuable means of treating chronic dysentery and diarrhœa with hepatic complications. Hence, it is one of the most frequent prescriptions of the author in the cases detailed, when considering the diseases of Europeans who have returned home after a long residence in India. He has used it in numberless cases, and believes it to be, on the ground of his experience, "an agent of the first order of importance."

We cannot avoid one more quotation; it is on one of the causes of chronic hepatic disease. It shows, what must have been observed already, the extreme care of the author to adapt remedies to the nature and stage of the disease. Having the utmost reliance upon the beneficial effects of mercury in some cases, in others he sees that only mischief can result from its use:

"It has been observed in Europe, that persons who have used mercury, either in the liberal and rapid manner or as a protracted alterative course, have frequently been afflicted subsequently with severe forms of hepatic disease. There can be no doubt that through the repeated use of this mineral, even when necessarily exhibited, the hepatic function is inordinately stimulated, to be proportionally enfeebled thereafter. The action of mercury on the liver may be viewed as having a close analogy to the primary excitement and subsequent exhaustion of the functions of the stomach produced by dram-drinking."

We are obliged to close our task, feeling that we have been unable to do Mr. Martin full justice in our remarks upon his valuable contribution to medical literature. Many subjects we have left entirely unnoticed; some of these are matters of interest only to Englishmen or Indians; others relate more immediately to the duties of naval and military surgeons; but there are others of deep interest to which we would gladly devote more attention, did we not fear to extend this article to too great a length. Sixty pages of the work are devoted to Epidemic Cholera, and nearly fifty more to Yellow Fever; the former we must leave for another opportunity, the latter for some one who can compare the description of the disease by the author with its appearance in our own borders. The chapters on Chronic Dysentery and Diarrhœa are especially valuable to the general practitioner; in fulness of detail, clearness of pathology, and scientific character of treatment proposed, they equal any other parts of the work.

Our task has been more to present the results of the author's observation and experience than to criticise his views; more to cull what he has learned during a life spent among a certain class of diseases, than to compare his doctrines with those of others who have written upon the same subjects. If, in accomplishing this task, we have imparted a tithe of the information and pleasure we have derived from perusing the volume, we shall be satisfied. It is a work which cannot fail to deeply interest every scientific physician, while it will prove a most valuable addition to the library of the naval and army surgeon.

